# Mini-review: Processed red meat intake and risk of neurodegenerative diseases

**DOI:** 10.3389/fnut.2025.1663647

**Published:** 2025-09-11

**Authors:** Ke-qian Chen, Wen-jin Cao, Zheng Liu, Ren-zhu Liu

**Affiliations:** Department of Clinical Pharmacy, Xiangtan Central Hospital, The Affiliated Hospital of Hunan University, Xiangtan, China

**Keywords:** processed red meat, neurodegenerative diseases, Alzheimer’s disease, Parkinson’s disease, Huntington’s disease, amyotrophic lateral sclerosis

## Abstract

Neurodegenerative diseases (NDDs) are a group of disorders characterized by the progressive loss of neurons in specific areas of the central nervous system. In recent years, more and more research has focused on the influence of diet on NDDs. As a common food, processed red meat is widely consumed worldwide. Many studies have shown that processed red meat may increase the risk of cancer, diabetes and cardiovascular disease. Unfortunately, it is unclear whether processed red meat affects NDDs. Therefore, we reviewed the existing literature on the role of processed meats in NDDs. We concluded that intake of processed meat may have an adverse effect on NDDs.

## 1 Introduction

The brain and spinal cord are composed of neurons. Neurons have different functions that affect human motor perception and memory cognition (1). Since neurons usually cannot be regenerated, excessive damage can severely impair brain and nerve function (2). NDDs are a group of disorders characterized by the progressive death of neurons, and they include Alzheimer’s disease (AD), Parkinson’s disease (PD), Huntington’s disease (HD), and Amyotrophic lateral sclerosis (ALS) (3).

Previous studies had focused on drugs and traditional plants in the treatment of NDDs (4, 5). With the development of society and medical technology, a large number of new treatment methods such as gene therapy, aquatherapy, brain energy rescue, nanoparticle therapy, and regenerative stem cell therapy have appeared (6–8). In addition to treatment, diet also plays an important role in NDDs. The mediterranean diet, the DASH (Dietary Approaches to Stop Hypertension) diet, and the MIND (Mediterranean-DASH Intervention for Neurodegenerative Delay) diet have been documented to protect against NDDs (9). Furthermore, some nutrients, such as vitamin B6, vitamin B12, folate, caffeine, and lecithin, have beneficial effects on NDDs (10).

**Figure 1:**
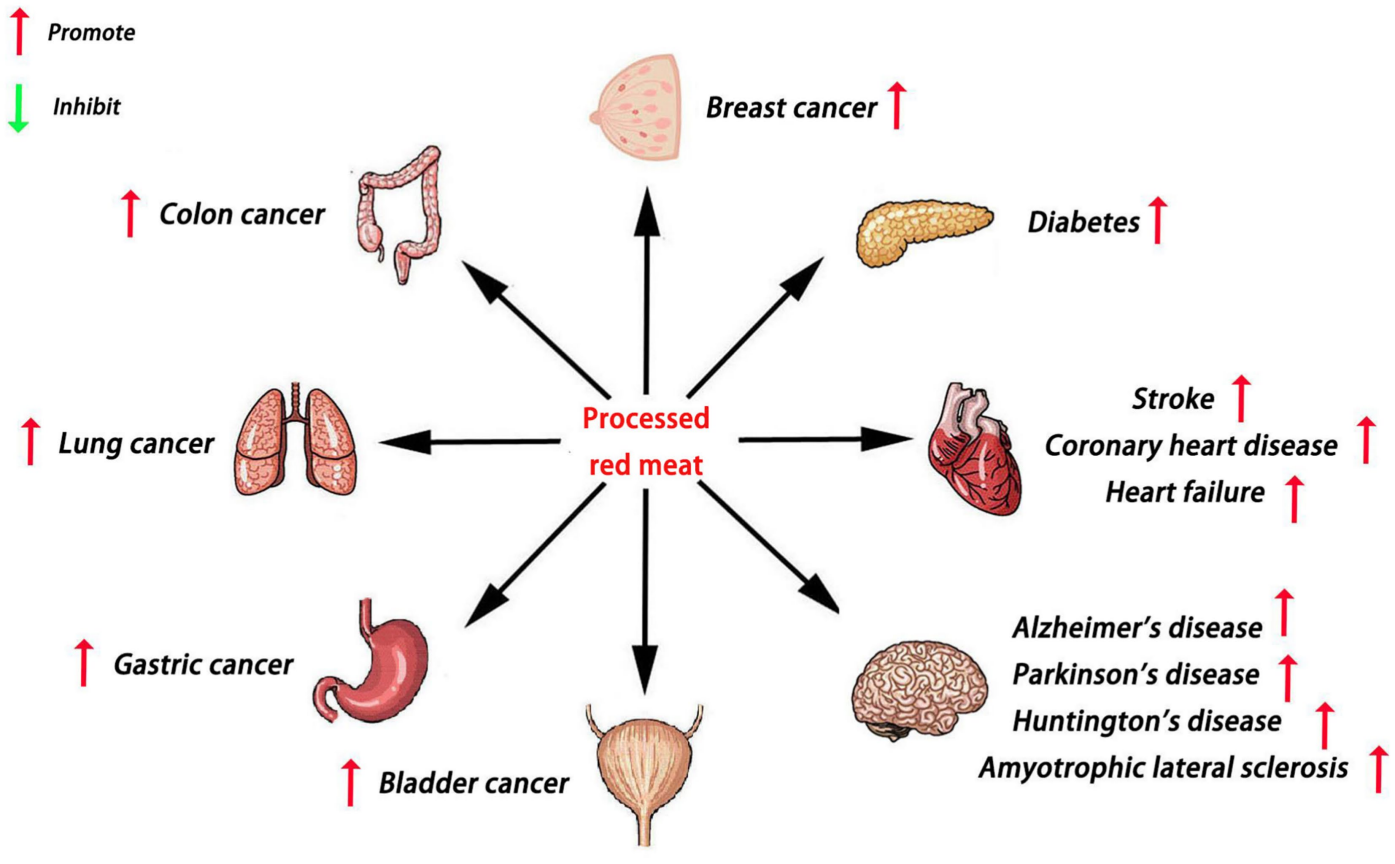
Main roles of processed red meat in various diseases.

Red meat is a type of meat that appears red before cooking, mainly including pork, beef, lamb, and other mammalian meat (11). As a popular food, processed red meat is consumed globally (12). However, many studies have reported that processed red meat may increase the risk of cancer, diabetes, and cardiovascular disease (13) (Figure 1). Regrettably, it is still unclear whether processed red meat influences NDDs. Therefore, we reviewed the current literature on the role of processed red meat in NDDs. We speculate that excessive intake of processed red meat may promote the development of NDDs.

As a motor neuron disease, ALS is primarily characterized by the loss of motor neurons in the brain and spinal cord (14). Pupillo et al. (15) surveyed 212 patients with newly diagnosed ALS from three Italian administrative regions. They found that processed red meat may be a risk factor for ALS. As the most common NDD, AD was first proposed by Alois Alzheimer in 1907 (16). Aggregation of Amyloid-β (Aβ) peptide is linked to the pathophysiology of AD (17). Numerous epidemiological studies have examined the relationship between the dietary heme intake (processed red meat) with the risk of AD (18, 19). Heme prevents the formation of Aβ peptide aggregates by binding with Aβ peptide (18). It may lead to dysfunctional mitochondria and altered metabolic activity in the brains of AD patients (20). Another study suggests that excessive consumption of processed red meat might correlate with the risk of mild cognitive impairment patients to develop AD (21). As the second most common NDD, PD has long been characterized by the loss of dopamine (DA) in the substantia nigra. Processed red meat may be one of the critical factors associated with an increased risk of PD (22). Zapała et al. (23) compared diet preferences in PD patients and healthy controls. They found that the consumption of processed red meat in PD patients was significantly higher than healthy controls (23). Coimbra CG’s study suggests that the elimination of red meat promotes the recovery of some motor functions in PD patients (24). Neuroinflammation and DNA damage are major mechanisms in PD pathogenesis (25). Several studies have shown that processed red meat consumption is positively linked to PD. Processed red meat consumption may promote inflammation (26). The chemical *N*-methyl-phenyl-tetrahydropyridine (MPTP) and 2-amino-1-methyl-6-phenylimidazo(4,5-b)pyridine (PhIP) are neurotoxicants formed in processed red meat (27). MPTP can cause a parkinsonian syndrome in men (28). PhIP can induce DNA damage in galactose-dependent SH-SY5Y cells (27).

Excessive consumption of processed red meat may increase the risk of diabetes, Alzheimer’s disease, Parkinson’s disease, stroke, coronary heart disease, heart failure, Huntington’s disease, amyotrophic lateral sclerosis, colon cancer, breast cancer, lung cancer, gastric cancer, and bladder cancer.

## 2 Mechanisms of NDDs

Over the past few decades, more and more NDDs have resulted in premature death or disability as the population ages (29). Understanding the pathogenesis of NDDs is important to explore the role of processed red meat in NDDs. Current studies have found that the pathogenesis of NDDs is related to oxidative stress, mitochondrial dysfunction, inflammation, and the disturbance of Ca^2+^ (30).

Oxidative stress is a state caused by the imbalance between reactive oxygen species production and antioxidant defense. It is characterized by excessive production of free radicals and reactive oxygen species (31). Studies have indicated that oxidative stress plays an important role in the pathogenesis of NDDs (32). Oxidative damage of nerve tissue has been found in NDDs such as AD, PD, HD, and ALS. On the one hand, a high concentration of ROS will damage the DNA, proteins, lipids, and other macromolecules in nerve cells, and eventually lead to neuronal necrosis and apoptosis. On the other hand, the use of free radical scavengers or antioxidants can significantly improve these NDDs (33).

Mitochondria are tiny structures in the cytoplasm that are involved in the production and metabolism of energy (34). Earlier studies have found that mitochondria are not only the main sources of ROS, but also the main “tools” for clearance (34). Mitochondrial dysfunction plays an important role in the pathogenesis of NDDs (35). Mitochondrial dysfunction can lead to the imbalance between ROS production and elimination, and ultimately lead to neuronal damage and apoptosis (35). The abundant vascular system in the brain guarantees the huge blood supply, and also provides sufficient glucose and oxygen for brain energy metabolism (36). Mitochondrial dysfunction can also contribute to the progression of NDDs by affecting energy metabolism (37).

It is well known that inflammation plays an important role in NDDs (38). The main sign of brain inflammation is the activation of glial cells. Under normal physiological conditions, microglia maintain the homeostasis of the central nervous system by engulfing pathogens and apoptotic cells (39). When microglia are repeatedly activated by inflammation, neuroinflammation is transformed into chronic inflammation and accompanied by the release of inflammatory factors such as IL-6, TNF-*α*, and IL-1. These inflammatory factors accelerate the production and aggregation of neurotoxic proteins, resulting in neuronal damage and death (39). Therefore, neuroinflammation is also a key mechanism in the pathogenesis of NDDs. As an important messenger in brain neurons, Ca^2+^ plays an important role in the development of neurons, the growth of axons, and the formation of synapses (40). When Ca^2+^ balance is disrupted, the growth and development of nerve cells are affected. On the one hand, excessive concentration of Ca^2+^ will increase the aggregation of Aβ protein and the over-phosphorylation of Tau protein, resulting in the impairment of patients’ learning and memory ability (40). On the other hand, excessive concentration of Ca^2+^ can also activate apoptosis pathways, aggravate oxidative stress, and cause apoptosis (40). Meanwhile, more and more studies have pointed out that the disturbance of zinc, iron, and copper can also lead to the occurrence of many NDDs (41).

In addition to the above mechanisms, pathogenic mechanisms of NDDs include the misfolding and aggregation of proteins, abnormal repair of DNA, excitatory toxins (glutamate), autophagy, pyroptosis, and ferroptosis (42–44).

## 3 Risks associated with components of processed red meat

### 3.1 Methionine

Processed red meat is a methionine-rich food (45). As an essential sulfur-containing amino acid, methionine is involved in various biochemical processes (46). Epidemiological studies have indicated that high methionine consumption has a negative effect on NDDs (47). Firstly, Methionine metabolism can produce toxic byproducts (homocysteine), which contribute to oxidative damage (48). Moreover, methionine-rich diet (processed red meat) can lead to mitochondrial dysfunction by impairing mitochondrial DNA integrity and affecting mitochondrial dynamics (47). Mitochondrial oxidative stress and dysfunction can contribute to NDD pathogenesis (35). Secondly, methionine-rich diet (processed red meat) has been shown to induce inflammation by activating pro-inflammatory signaling pathways and generating inflammatory mediators (47). Studies have shown that inflammation can contribute to NDD pathogenesis (49). Thirdly, the health of the microvasculature, blood-brain barrier, proteostasis, and functional connectivity are essential for efficient cognitive function (50). Methionine-rich diet (processed red meat) can lead to cognitive impairments and neuronal damage by disrupting these normal processes (47).

### 3.2 Iron

Processed red meat is rich in heme iron (51). As an important cofactor, iron is essential for neuronal development, synaptic plasticity, and myelination. However, excessive intake of iron can be harmful to health (52). Studies have shown that high consumption of processed red meat and its products, and thereby iron, particularly in the form of heme, increases the risk of many diseases (53). For decades, deposits of iron have been detected in patients with AD, PD, ALS, and HD (54). Excessive iron accumulation is harmful because it can promote the formation of free radicals resulting in oxidative stress, lipid peroxidation, protein aggregation, and eventually cell/neuronal death (55).

### 3.3 Sodium

Processed red meat is also a high-sodium food (56). However, the relationship between the high-sodium diet and AD is not clear. The alteration of sodium homeostasis significantly contributes to synaptic dysfunction and neuronal loss in AD (57). Attenuation of hippocampal hyperactivity, an earliest neuronal abnormality observed in AD brains, has been attributed in part to the dysfunction of sodium channels (58). Unusual cerebrovascular morphology and structure may contribute to cerebral hypoperfusion in AD. Baumgartner et al. (58) found that a high-sodium diet reduced vascular density (59). These results suggest that a high-sodium diet can induce cerebrovascular morphology changes in AD mouse models (59). In addition, Taheri et al. (60) found that a high-sodium diet influences the accumulation of Aβ peptide, exacerbates cognitive decline, and increases the propensity to AD. It is well known that the pathophysiology of HD is very complex. Intracerebral sodium accumulation has a crucial role in the pathophysiology of HD (61). Interestingly, Reetz et al. (62) found an increase in sodium concentration of the entire brain in HD patients.

### 3.4 Nitrite and nitrate

Boll et al. (63) recorded the sum of nitrites and nitrates from patients with any of the four NDDs (PD/AD/HD/ALS), and they found it increased in all of them. As a metabolite of nitric oxide (NO), nitrite can lead to nitrosative stress in the nigrostriatal system (64). There is evidence that nitrosative stress is an important factor promoting degeneration in PD (65). 3-nitropropionic acid (3-NP), a hemotoxin of fungal origin, has been used in rodents to model HD (66). A large number of studies have shown that 3-NP can significantly increase the levels of nitrite in HD models (67, 68). Many natural drugs such as rutin, lycopene, resveratrol, lutein, and safranal can prevent 3-NP-induced HD by decreasing the levels of nitrite (69–73). Previous studies have demonstrated that the nitrite and nitrate levels were significantly increased in the cerebrospinal fluid (CSF) and serum from ALS patients (74). Interestingly, motoneuron survival was inversely correlated with nitrate/nitrite concentrations in the ALS mouse model (75). A systemic pro-inflammatory state plays a central role in ALS pathogenesis (76). As the macrophages of the central nervous system, microglia are responsible for the inflammatory component of ALS (77). Meanwhile, microglia also contribute to motoneuron injury in ALS. In the pathogenesis of ALS, microglia can induce more neuronal death by producing and releasing more nitrite and nitrate (78).

### 3.5 Phosphatidylcholine

**Figure 2:**
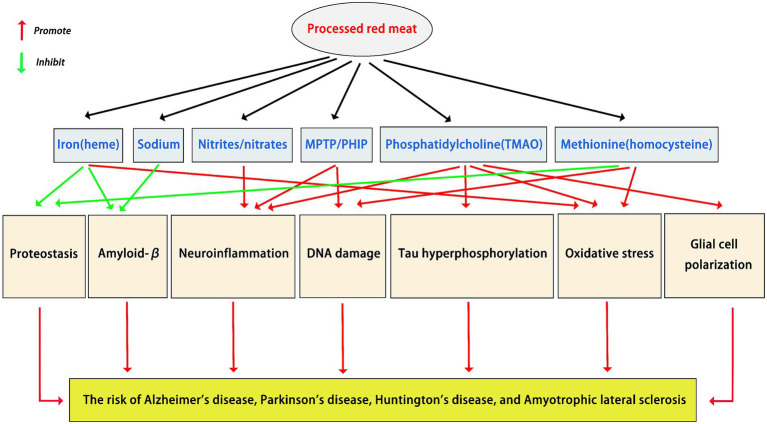
The mechanisms of processed red meat components in neurodegenerative diseases.

Extensive data demonstrate that lipids play a crucial role in NDD pathogenesis. For example, the accumulation of lipids is a risk factor for PD (79). Lipid dysregulation is a feature of ALS (80). Aberrant lipid metabolism is linked to the pathophysiology of AD (81). Phosphatidylcholine is one of the most common fats found in processed red meat (82). Some studies suggest that the consumption of phosphatidylcholine may have adverse effects on NDDs. As an early marker of neurodegeneration, phosphatidylcholine may promote tau hyperphosphorylation (83). Trimethylamine n-oxide (TMAO) is a gut microbiota metabolite derived from phosphatidylcholine (84). There is evidence that TMAO is associated with the pathogenesis of various NDDs (85, 86). On the one hand, TMAO levels increase with age-related cognitive dysfunction (86). On the other hand, TMAO also induces mitochondrial dysfunction, oxidative stress, neuroinflammation, and glial cell polarization in the brain (85). The mechanisms of processed red meat components in NDDs are shown in Figure 2.

## 4 Discussion

With the development of society and the economy, more and more unhealthy dietary patterns have harmful effects on people’s brains (85). Among these dietary patterns, processed red meat consumption is an interesting potential factor. Accumulating evidence suggests that processed red meat intake might be associated with NDD pathogenesis. The potential adverse effects on NDDs of processed red meat have been attributed to its ingredients such as methionine (47), heme iron (54), sodium (57), nitrite/nitrate (63), and phosphatidylcholine (83). Many studies have revealed the harmful effects of these ingredients on brain health (23, 87–89). Nevertheless, these studies have several limitations (23, 87–89). Firstly, the sample size of many studies was not large enough to ensure sufficient statistical power (88). Meanwhile, some cases may not have been classified by type of disease, which may attenuate association between processed red meat intake and risk of NDD development (89). Secondly, harmful substances of processed red meat may also be produced during the cooking of other foods (83). Meanwhile, some components of processed red meat play a protective role in NDDs (83). Therefore, it is difficult to conclude that processed red meat is the main cause of NDDs. There may be an intricate influence of multiple factors, including alcohol consumption, smoking, obesity, and stress (83). Thirdly, the conflicting results of some studies may be due to the fact that the dose of processed red meat is not enough (87). In the future, different dosage standards will need to be used when we study the relationship between processed red meat and NDDs. Meanwhile, it would be of interest to go beyond the use of questionnaires by also including biomarkers or metabolomics to study the associations between processed red meat consumption and NDDs. In addition, some of the observed results in previous studies may be limited by inadequate adjustment for potential confounders (23). More complete adjustment for a broad spectrum of potential confounders in future studies could help to address this potential limitation. Nevertheless, we believe that the consumption of high red meat may have adverse effects on NDDs. In conclusion, it is interesting to explore the relationship between the processed red meat and NDDs. Understanding the mechanism and role of processed red meat in NDDs has broad prospects for the prevention and treatment of NDDs.
